# Waldenstrom macroglobulinemia: prognosis and management

**DOI:** 10.1038/bcj.2015.28

**Published:** 2015-03-27

**Authors:** A Oza, S V Rajkumar

**Affiliations:** 1 Department of Medicine, Indiana University School of Medicine, Indianapolis, IN, USA; 2 Division of Hematology, Mayo Clinic, Rochester, MN, USA

## Abstract

Waldenstrom macroglobulinemia (WM) is a B-cell lymphoplasmacytic lymphoma characterized by monoclonal immunoglobulin M protein in the serum and infiltration of bone marrow with lymphoplasmacytic cells. Asymptomatic patients can be observed without therapy. First-line therapy should consist of the monoclonal anti-CD20 antibody, rituximab, given typically in combination with other agents. We prefer dexamethasone, rituximab, cyclophosphamide (DRC) as initial therapy for most patients with symptomatic WM. Other reasonable options are bortezomib, rituximab, dexamethasone (BoRD) or bendamustine plus rituximab (BR). All of these regimens are associated with excellent response and tolerability. Initial therapy is usually administered for 6 months, followed by observation. Response to therapy is assessed using the standard response criteria developed by the International Working Group on Waldenstrom macroglobulinemia. Relapse is almost inevitable in WM but may occur years after initial therapy. In symptomatic patients relapsing more than 1–2 years after initial therapy, the original treatment can be repeated. For relapse occurring sooner, an alternative regimen is used. In select patients, high-dose chemotherapy followed by autologous hematopoietic cell transplantation may be an option at relapse. Options for therapy of relapsed WM besides regimens used in the front-line setting include ibrutinib, purine nucleoside analogs (cladribine, fludarabine), carfilzomib and immunomodulatory agents (thalidomide, lenalidomide).

## Introduction

Waldenstrom macroglobulinemia (WM) is defined as a B-cell lymphoplasmacytic lymphoma, characterized by monoclonal immunoglobulin M protein in the serum and infiltration of bone marrow with lymphoplasmacytic cells.^1^ A majority of patients with WM have a recurrent mutation of the MYD88 gene (MYD88 L265P).^2, 3^ The highest incidence of WM occurs among older individuals, with a median age at diagnosis in the 60s.^1, 4^ Although approximately 25% of patients are asymptomatic at the time of diagnosis, most patients present with symptoms attributable to tumor burden, including anemia, pancytopenia, organomegaly, neuropathy, amyloidosis, cryoglobulinemia, night sweats and symptomatic hyperviscosity.^5, 6, 7^ The focus of this paper is on the prognosis and treatment of WM.^8, 9^

## Prognosis

WM is a fairly indolent, chronic disease in most patients. The median survival has varied in studies, from 5 years to nearly 11 years.^10^ The main causes of death because of WM include disease progression, transformation to high-grade lymphoma or complications of therapy. However, owing to the advanced age of these patients, many will die of unrelated causes.^5^ Mortality is linked to the development of symptoms; the mortality of asymptomatic patients is similar to that of the general population, whereas it is significantly higher in symptomatic patients.^11, 12^ No studies have demonstrated a survival benefit of treating asymptomatic patients, nor are there data to suggest delaying therapy until symptoms develop adversely affects response to treatment.^1, 11, 13^ Furthermore, following a surveillance approach can maintain the patient's quality of life, and limit exposure to chemotherapy and its potential side effects.^8^ The decision between surveillance and treatment remains a clinical one; however, use of prognostic models may help guide the decision between more aggressive therapy vs avoidance of therapy-related complications and preservation of quality of life.^1, 9^

Several staging systems have been proposed to risk stratify patients with WM and to aid in prognosis (Table 1).^10, 14, 15^ Dhodapkar and colleagues^14^ developed a three-parameter staging system for WM based on the results of a multicenter clinical trial conducted by the Southwest Oncology Group. This model uses hemoglobin concentration, β2-microglobulin levels and serum immunoglobulin (Ig) M level to classify patients into four prognostic groups with significantly different 5-year survival rates. As the model was developed in the setting of a clinical trial, it is unclear how prognosis would differ for patients who are not candidates for clinical trials including patients with poor performance status. On the basis of another study of 337 symptomatic patients with WM, a prognostic model was created at the Mayo Clinic consisting of age >65 and presence of organomegaly.^15^ Having neither of these factors conferred a 10-year estimated survival rate of 57%. One factor was associated with 16% 10-year survival, and the presence of both factors was associated with 5% survival at 10 years. The addition of elevated β2-microglobulin ⩾4 mg/l was associated with a threefold increased risk of death. Of note, the prognostic significance of serum IgM levels and organomegaly has varied in different studies, whereas age is consistently a poor prognostic indicator.

Given the wide selection of new treatments available for WM, a standardized scoring system has been developed recently, known as the International prognostic scoring system for Waldenstrom macroglobulinemia (IPSSWM), to help guide treatment based on prognosis in symptomatic patients^10^ (Table 1). Using multivariate analysis, five variables were identified which were associated with adverse outcomes: age >65, hemoglobin ⩽11.5, platelet ⩽100 000/microliter, β_2_–microglobulin >3 mg/l and serum monoclonal protein concentration >70 g/l. The low-risk group is defined as having no more than one of these characteristics, excluding age >65, and is associated of an average 5-year survival of 87%. Intermediate risk patients have two of the above characteristics or age >65, and is associated with 5-year survival of 68% on average. Finally, the high-risk group has three or more adverse characteristics, with a 5-year survival of 36%.

On the basis of a prospective study of 72 patients, von Willebrand factor antigen level was identified as a prognostic factor in WM.^16^ High levels were associated with poor prognosis that did not improve with disease control. Low levels on the other hand were associated with increased bleeding risk but improved with lowering of serum IgM levels.

## Treatment

WM is incurable with currently available therapies. Treatment is instead focused on symptom-control and prevention of end-organ damage.^1, 8, 9^ There is no standard therapy for WM, but rather various drugs that have been shown to be effective either alone or in combinations (Tables 2 and 3).^17, 18, 19, 20, 21, 22, 23, 24, 25, 26, 27^ Asymptomatic patients should be followed with surveillance, while symptomatic patients should be considered for treatment. The severity of symptoms dictates the intensity of the treatment regimen, however, several factors, such as age, cytopenias, need for quick disease control and potential candidacy for stem cell transplant, should be considered. Care should be given to avoid treatments that may compromise stem-cell collection in patients who are candidates for autologous hematopoietic cell transplantation (HCT).

### Treatment of hyperviscosity

Hyperviscosity is a clinical emergency. Manifestations are the result of shear forces which cause venous damage, and include visual changes resulting from retinal flame-shaped hemorrhages, epistaxis, gingival bleeding, headaches and dizziness; more severe manifestations include stupor and coma. Hyperviscosity syndrome occurs when the serum viscosity is >4 (viscosity of water is 1), which corresponds to serum IgM of at least roughly 4000 mg/dl. Immediate management is the removal of IgM from systemic circulation, via plasmapheresis. Transfusion with red blood cells should be avoided prior to plasmapheresis to avoid increasing serum viscosity. Although there are no randomized trials on the use of plasmapheresis in managing hyperviscosity, total plasma exchange (roughly 3–4 l in an adult, replaced with albumin rather than plasma) is repeated daily until normal serum viscosity is achieved and symptoms are relieved. Plasmapheresis does not alter the disease course, therefore, after plasmapheresis is complete, chemotherapy should be initiated. This approach also helps avoid potential loss of drugs that may be bound to protein in the serum.

### Single-agent therapy

#### Rituximab

As all WM cells express CD-20, it is generally agreed upon that first-line therapy should consist of monoclonal anti-CD20 antibody, rituximab, alone or preferably in combination. ^9^ As it may take several months to achieve the maximum response to rituximab, it is a poor choice as single-agent therapy in patients in whom a rapid response is needed, or in patients with IgM levels above 3 g/dl. However, in selected patients, it has been shown to reduce neurologic symptoms, improve cytopenias and bone marrow involvement, while still being fairly well tolerated without damaging stem cells.^17, 28, 29, 30^ Of note, there may be a paradoxical increase in monoclonal protein levels after starting rituximab therapy, termed the 'rituximab flare' which may persist for up to 4 months.^8^ This does not indicate treatment failure, but plasmapheresis may be necessary to avoid the development of hyperviscosity. A prospective trial randomized 69 patients with lymphoplasmacytic lymphoma, 48 of whom met criteria for WM, to receive rituximab, cyclophosphamide, doxorubicin, vincristine, prednisone (CHOP) vs rituximab plus CHOP (R-CHOP).^18^ The R-CHOP group as a whole had significantly higher overall response rate as compared with the CHOP group, (94 vs 67%), as well as in the WM subgroup (91 vs 60%). R-CHOP was also associated with a significantly longer time to treatment failure (median 63 months vs 22 months) which also carried over to the WM subgroup. No major difference in treatment-associated toxicity was observed between the two groups. A phase II trial of single-agent rituximab in 69 patients with WM (34 untreated, 35 previously treated) was undertaken, which showed an objective response in 27.5% and minor response in 24.6% of patients.^17^ Single-agent rituximab is a reasonable treatment option for minimally symptomatic, low-risk patients with only moderate hematologic compromise (hemoglobin 10–11 g/dl or platelets 100 000–120 000/l), as well as patients with IgM-associated neuropathy, or hemolytic anemia which is refractory to corticosteroids.^8^ Rituximab plus additional agents is preferred for patients with severe cytopenias (hemoglobin <10 g/dl or platelets <100 000/l), constitutional symptoms or symptoms of hyperviscosity. Although the combination of rituximab plus another agent has been shown to attain a high response rate more rapidly, it is unclear whether it confers prolonged survival. In patients who are rituximab-naïve and respond to rituximab-containing regimens, maintenance rituximab has been shown to improve overall and progression-free survival, although it is also associated with higher rates of infection, and is generally not recommended.^1, 8, 31^

#### Bortezomib

The Waldenstrom Macroglobulinemia Clinical Trials Group (WMCTG) conducted a multicenter study investigating the use of bortezomib, a reversible proteasome inhibitor, in the management of WM.^32^ The results of this study showed an overall response rate of 85%, with median duration to response of only 1.4 months. The most common side effect was sensory neuropathy, which resolved or improved in almost all patients after cessation of therapy. Of note, discordance between serum IgM levels and bone marrow response has been observed in bortezomib therapy, necessitating bone marrow biopsy or computed tomography scans to monitor response to therapy.^33^

#### Alkylating agents

Of the alkylating agents, oral chlorambucil is the most commonly used agent, resulting in at least a partial response in >50% of patients.^6^ Therapy continues until symptoms resolve and serum IgM concentration plateaus. Alkylating agents have been associated with the development of secondary leukemias and myelodysplasia. Chlorambucil should be avoided in patients who are considered candidates for stem cell transplantation.

### Combination therapy

**Figure 1:**
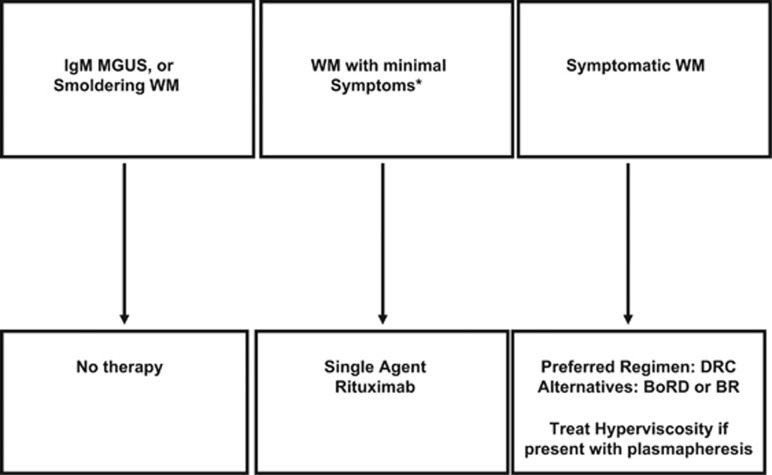
Approach to the treatment of newly diagnosed WM. MGUS, monoclonal gammopathy of undetermined significance; WM, Waldenstrom Macroglobulinemia; DRC, dexamethasone, rituximab, cyclophosphamide; BoRd, bortezomib, rituximab, dexamethasone; BR, bendamustine, rituximab.

Multiple rituximab-containing regimens, which lack stem-cell toxicity, are available: dexamethasone, rituximab, cyclophosphamide (DRC); bortezomib, rituximab with or without dexamethasone; bendamustine with rituximab (BR). We prefer any of these three regimens for front-line therapy (Figure 1). The primary factors influencing selection among the available regimens are drug toxicities and individual patient characteristics. For example, in a patient with peripheral neuropathy, DRC may be preferred because of the high incidence of neuropathy associated with bortezomib. In patients with bulky disease, BR may be preferred for rapid control. The major studies supporting the use of these and other commonly used combinations in WM is presented below.

#### Dexamethasone, rituximab and cyclophosphamide (DRC)

In a phase-II trial of symptomatic, treatment-naïve patients with WM, DRC was associated with an 83% overall response rate and 7% complete response rate.^21^ Two-year overall survival was 81%, with progression-free survival of 67%. The toxicity profile was mild, mainly consisting of nausea, alopecia, neutropenia and mild infusion reactions to rituximab.

#### Bortezomib, rituximab and dexamethasone

In two separate phase-II trials of symptomatic, previously untreated patients with WM, BRD was associated with 85%^34^ and 96%^20^ overall response rates, respectively. However, in one of the studies, there was a 27% discontinuation rate because of drug toxicity.^34^ The most common toxicity reported was peripheral neuropathy, occurring in 46% of patients in the study by Dimopoulos and colleagues,^34^ and 69% in the study conducted by Treon and colleagues.^20^ In patients with myeloma, bortezomib neuropathy has been markedly abrogated by the use of weekly subcutaneous bortezomib rather than the twice weekly intravenous schedule.^35, 36, 37^ On the basis of these data, we recommend weekly subcutaneous bortezomib for patients with WM as well, similar to current recommendations in myeloma.^38^

#### Bendamustine plus rituximab

Bendamustine is a nitrogen mustard which bears structural similarities to alkylating agents as well as purine analogs. A phase II trial of BR in 63 patients (including 17 with WM/lymphoplasmacytic lymphoma) with relapsed/refractory low-grade or mantle cell lymphoma showed overall response rate of 90% and complete response rate of 60%.^19^ Median progression-free survival was 24 months. The most common side effect was leukopenia, with grade 3 and 4 severity occurring in 16%. Treon *et al.*^39^ studied the outcome of 30 patients with relapsed/refractory WM treated with BR. The overall response rate (very good partial response and partial response) was 83.3% including some patients who were intolerant to rituximab and received bendamustine alone. Treatment was generally well-tolerated although prolonged myelosuppression was noted in some of the patients who had previously received nucleoside analog therapy. A prospective study of previously untreated indolent Non-Hodgkin lymphoma/Mantle Cell lymphoma randomized patients to receive BR or R-CHOP.^40^ Among the 41 WM patients included in the study, both arms showed 95% response rates, with longer progression-free survival in the BR arm vs R-CHOP (69.5 months vs 28.1). There were also lower rates of adverse events observed in the BR arm vs R-CHOP.

### Purine nucleoside analogs

Nucleoside analogs have significant activity in the treatment of WM. However, these agents should be avoided as initial therapy in patients who may be candidates for HCT as they are toxic to stem cells and may compromise the ability to harvest stem cells for transplant.

Fludarabine and cladribine are two purine nucleoside analogs, which have been found to be effective in WM, either alone or in combinations.^41, 42, 43^ The combination of purine nucleoside analogs with rituximab improves response rates with little added toxicity, and may be ideal especially when rapid disease control is needed.^23, 44^ A phase III trial comparing chlorambucil and fludarabine in treatment of >300 patients with WM showed improved progression-free survival and longer duration of response with fludarabine use.^45^ As a single agent, fludarabine is associated with > 50% survival at 8 years in patients with one risk factor, and >30% survival in those with two risk factors.^14^ Rituximab plus fludarabine has an overall response rate of >95%. Severe toxicities associated with fludarabine include neutropenia and thrombocytopenia, as well as a future predisposition to develop myelodysplasia and lymphomas/leukemias.^23, 46^ Alternatively, combination cladribine with rituximab was associated with similar overall response rates (90%), but was generally better-tolerated producing milder cytopenias.^24^ An Italian study of 43 patients with symptomatic WM treated with fludarabine/cyclophosphamide/rituximab showed an overall response rate of 79%.^22^ Severe neutropenia (grade 3–4) was seen in 88% of patients, but was prolonged in 44% of patients.

### Other drugs

#### Alemtuzumab

Alemtuzumab is a monoclonal antibody against CD52, a glycoprotein found on WM lymphoplasmacytic cells as well as mast cells, and stimulates growth and survival of WM lymphoplasmacytic cells. WMCTG conducted a multicenter study using alemtuzumab to treat 27 patients with WM.^47^ Overall response rate was 75%, with cytopenias occurring commonly among patients who had been treated previously.

#### Lenalidomide

Immunomodulatory agents have been combined with monoclonal antibodies in an effort to improve disease response in WM while avoiding the side effects of chemotherapy. Two such agents are thalidomide, and lenalidomide, its more potent derivative. The combination of lenalidomide with rituximab was studied in 16 symptomatic patients with WM who were naïve to both agents and 12 of whom were previously untreated.^48^ The overall response rate was 50%, with a minor response rate of 25%, both inferior to a study of thalidomide/rituximab by the same authors.^27^ The median time to progression among the responders was 18.9 months. Anemia also developed in >80% of the study patients, despite dose reductions of lenalidomide, and resulted in premature discontinuation and cessation of further enrollment. Hematocrit levels improved after lenalidomide cessation in the 12 evaluable patients.

#### Thalidomide

A phase-II trial of thalidomide with rituximab in patients with symptomatic WM was undertaken in 35 patients who had not been treated with either drug previously.^27^ Results showed an overall response rate of 72%. Neuropathy was a major side effect, necessitating dose reductions in all patients in the trial.

#### Ibrutinib

Ibrutinib is an irreversible and selective inhibitor of Bruton's tyrosine kinase, a signaling molecule in the B-cell antigen receptor cascade which has been implicated in many B-cell malignancies. Advani *et al.*^49^ studied the outcome of ibrutinib therapy in 56 patients with relapsed/refractory B-cell malignancies, including 4 with WM. Among the 50 evaluable patients, a 60% response rate was observed (partial response and complete response), with median progression-free survival of 13.6 months in all patients. The maximum-tolerated dose was not reached, and no dose-limiting adverse events were observed. Most adverse events were low-grade (1 or 2) and easily manageable if not reversible. In a separate phase II study, 63 patients with symptomatic relapsed/refractory WM were treated with ibrutinib.^26^ Overall investigator-assessed response rate was 81%, while 57% achieved at least a major response. The response rate as assessed by an Independent Review Committee was 62% (95% confidence interval, 48.8, 73.9). The median time to achieve a response was 1.2 months. The most common side effects with ibrutinib are low blood counts, gastrointestinal symptoms, rash, muscle spasms and fatigue. Ibrutinib was approved by the Food and Drug Administration for the treatment of WM in 2015.

#### Carfilzomib

Carfilzomib is a proteasome inhibitor, which unlike bortezomib, is considered neuropathy-sparing. A phase II trial of 31 patients with symptomatic WM, previously treated with rituximab or bortezomib, were treated with carfilzomib, rituximab and dexamethasone.^25^ Overall response rate of 87% was observed. The most common toxicities included hyperglycemia secondary to dexamethasone, and elevated serum lipase, both of which were asymptomatic and reversible. Grade 1–2 peripheral neuropathy was seen in 19% of patients.

## Approach to initial therapy

There are no good data from randomized trials to guide the choice of initial therapy in WM among modern regimens. Initial therapy for WM is usually given for approximately 6 months, and then patients are observed. We prefer DRC as the initial treatment regimen as it is convenient, effective and does not compromise the ability to collect stem cells (Figure 1).^8^ Two reasonable alternative options would be bortezomib, rituximab, dexamethasone and BR. The choice of initial therapy varies according to cost and drug availability in various countries. In WM patients with mild anemia as the main criterion for initiating therapy who have IgM levels less than 3 gm/dl, single-agent rituximab may be a reasonable option for initial therapy.^8, 17^ However, it will take many months for a decrease in IgM levels to occur with rituximab alone. Ibrutinib has been recently approved for the treatment of WM in the United States, but there are limited data in the front-line setting. In patients who are not considered candidates for transplantation, additional options include a purine nucleoside analog such as cladribine.

## Response to therapy

Many of the initial studies on treatment of WM used different criteria for response to therapy, making it difficult to compare results. A standardized response criterion to measure the effect of treatment has been created by the International Working Group on Waldenstrom macroglobulinemia.^50, 51^ Patients are evaluated prior to each cycle of treatment to assess response to therapy. Complete response is defined as the disappearance of serum and urine monoclonal protein (assessed by immunofixation), normal serum IgM level, resolution of lymphadenopathy and organomegaly, lack of histologic bone marrow involvement, and absence of signs and symptoms attributable to WM. This must be confirmed 6 weeks later with a second immunofixation. Very good partial response is at least 90% reduction in serum IgM, resolution of lymphadenopathy and organomegaly, and resolution of symptoms. Partial response requires between 50 and 90% reduction in serum monoclonal IgM, at least 50% reduction in adenopathy and organomegaly, with no new signs or symptoms of active disease. Minor response requires between 25 and 50% reduction in serum monoclonal IgM without new signs or symptoms of active disease. Progressive disease is defined as two measurements showing at least 25% increase in serum monoclonal IgM, or progression of anemia/thrombocytopenia/leukopenia, adenopathy/organomegaly or increase in symptoms attributed to WM. Finally, stable disease are those which do not meet the criteria for minor response or progressive disease. 'Major response' includes complete response, very good partial response or partial response. Resolution of organomegaly must be confirmed by imaging. After completion of treatment, patients should undergo surveillance every 3 months for the first year, then every 6 months. This evaluation should include a complete history and physical exam, complete blood count, and measurement of monoclonal IgM in the serum or urine. Imaging should only be obtained if indicated by symptoms.

## Management of relapsed disease

Relapse is associated with an increase in the IgM monoclonal protein in the serum along with associated symptoms of WM. In many patients, the IgM protein level may not be as high at relapse as it was at the time of initial diagnosis, even when the tumor burden and symptoms are similar.^8^ Unfortunately, given that WM is incurable, almost all patients will relapse after initial therapy.

**Figure 2:**
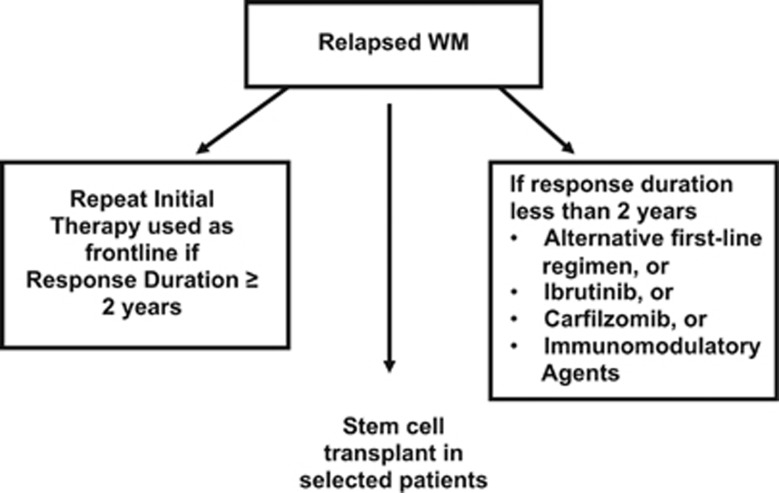
Approach to the treatment of relapsed Waldenstrom Macroglobulinemia. WM, Waldenstrom Macroglobulinemia.

In patients who achieve an initial major response, the decision to resume therapy should be based on recurrence of cytopenias or symptoms, rather than monoclonal protein levels alone. Type of therapy used at the time of relapse is determined by the response to initial therapy (Figure 2). In symptomatic patients relapsing >1–2 years after initial therapy, the original treatment can be repeated. On the other hand, for relapse occurring <1–2 years after initial treatment, an alternative regimen is used. The regimens listed on Table 2 can be tried in a sequential manner as appropriate. Besides regimens used in the front-line setting, the other options for therapy in previously treated patients include ibrutinib, lenalidomide and chlorambucil.

### Stem cell transplantation

Select patients, who have good performance status and whose disease is chemo-sensitive at the time of transplant, may be candidates for high-dose chemotherapy followed by autologous HCT at first relapse.^52^ Studies have shown good outcomes with autologous HCT in previously treated and treatment-naïve patients.^53^ In the European Group for Blood and Marrow Transplantation (EBMT) study, autologous HCT was generally well tolerated, with non-relapse mortality of approximately 4% at 1 year. ^54^ The 5-year progression-free and overall survival rates were 39.7% and 68.5%, respectively. Similar results have been reported by other groups.^9^ Although good outcomes can still be seen in patients who have received multiple previous lines of therapies, and those who are considered refractory to therapy at the time of transplant, both of these factors do adversely affect expected outcomes.

Allogenic stem cell transplant has also been evaluated.^55^ In a study of 86 patients by the Lymphoma Working Party of the EBMT, the non-relapse mortality at 3 years was 33% for myeloablative and 23% for non-myeloablative allogeneic transplantation. The 5-year progression-free and overall survival rates were 56% and 62%, respectively, for myeloablative transplantation and 49% and 64%, respectively, for non-myeloablative transplantation. At present, it is preferable to consider allogeneic transplantation in patients with WM mainly in the setting of clinical trials.

## Future directions

Major advances have occurred in the diagnosis and treatment of WM, including several new options for therapy. We now need comparative clinical trials to determine the optimal choice of therapy in the front-line setting, role of transplantation and the sequence of treatments. For example, the approval of ibrutinib for WM is exciting, but we need studies in the front-line setting to determine the role of this agent relative to other commonly used regimens in WM. As WM generally has a chronic indolent course, care should be exercised in picking treatments that do not disrupt quality of life. It is therefore important that future studies compare treatment options using patient-reported quality of life outcome measures in addition to standard metrics of depth and duration of response. Finally, we need additional studies to determine the role of the MYD88 L265P mutation in pathogenesis, especially with regard to the role in transformation of IgM MGUS to WM.

## Figures and Tables

**Table 1:** Prognostic staging systems in Waldenstrom macroglobulinemia

*Staging system*	*Prognosis*
Southwest Oncology Group^10^	5-year OS
Stage A (low risk): β2-microglobulin <3 mg/dl and Hgb ⩾120 g/l	87%
Stage B (medium risk): β2-microglobulin <3 mg/l and Hgb <120 g/l	63%
Stage C (medium risk): β2-microglobulin >3 mg/l and serum IgM ⩾40 g/l	53%
Stage D (high risk): β2-microglobulin ⩾3 mg/l and serum IgM <40 g/l	21%
	
Mayo Clinic^14^	10-year OS
*Risk factors: Age >65 and presence of organomegaly*	
No risk factor	57%
Any one risk factor	16%
Both risk factors	5%
	
International prognostic scoring system for Waldenstrom macroglobulinemia (IPSSWM)^15^	10-year OS
*Risk factors: age > 65, hemoglobin ⩽115 g/l, platelet ≤ 100 000/microliter, β*_*2*_*–microglobulin >3 mg/l and serum monoclonal protein concentration >70 g/l*	
None or one risk factor (excluding age >65)	87%
Any two risk factors or age >65	68%
Any 3 or more risk factors	36%

Abbreviation: OS, overall survival.

**Table 2:** Response to therapy with common treatment regimens in Waldenstrom macroglobulinemia

*Regimen*	*Disease/treatment status*	*Overall response rate*	*Complete response rate*
Rituximab^17^	Untreated and previously treated	52.2%	0%
R- CHOP^18^	Untreated	94% (91% in WM group)	9%
Bendamustine/rituximab^19^	Relapsed/refractory	90%	60%
BoRD^20^	Untreated	96%	13% (8.7% near-complete responses)
DRC^21^	Untreated	83%	7%
FCR^22^	Untreated and previously treated	79%	11.6%
Fludarabine/rituximab^23^	<2 prior therapies	95.3%	4.7%
Rituximab/cladribine^24^	Untreated and previously treated	89.6%	24.1%
CaRD^25^	Untreated with rituximab or proteasome inhibitor	87.1%	3.2%
Ibrutinib^26^	Relapsed/refractory	57.1%	0%
Thalidomide/rituximab^27^	Untreated and previously treated	64%	4%

Abbreviations: BoRd, bortezomib, rituximab, dexamethasone; BR, bendamustine, rituximab; CaRD, carfilzomib, cyclophosphamide, dexamethasone; DRC, dexamethasone, rituximab, cyclophosphamide; FCR, fludarabine, cyclophosphamide, rituximab; R-CHOP, Rituxan, cyclophosphamide, doxorubicin, vincristine, prednisone; WM, Waldenstrom macroglobulinemia.

**Table 3:** Dosing and adverse events of common regimens used in the treatment of Waldenstrom macroglobulinemia

*Regimen*	*Dosing schedule*	*Main reported side effects*
Rituximab^17^	Rituximab 375 mg/m^2^ IV weekly × 4 weeks	Infusion-related reactions, increased risk of infections
R- CHOP^18^	Rituximab 375 mg/m^2^ on day 1 Cyclophosphamide 750 mg/m^2^ IV on day 1 Doxorubicin 50 mg/m^2^ IV on day 1 Vincristine 1.4 mg/m^2^ (max. 2.0 mg/day) IV on day 1 Prednisone 100 mg/m^2^ oral on days 1–5 Repeated every 3 weeks × 4–8 cycles (At signs of neuropathy, application of vincristine was stopped in the subsequent cycles)	Granulocytopenia of grades 3 and 4 in 72% alopecia, nausea and vomiting
Bendamustine/rituximab^19^	Bendamustine IV 90 mg/m^2^ on days 1 and 2 Rituximab IV 375 mg/m^2^ on day 1 Maximum 4 cycles every 4 weeks	Myelosuppression, 16% grade 3–4 leukopenia, 3% grade 3–4 thrombocytopenia
BoRD^20^	Bortezomib 1.3 mg/m^2^ subcutaneous once a week^a^ Dexamethasone 40 mg IV or oral on days 1, 8, 15, 22 Rituximab 375 mg/m^2^ IV on day 11 Repeat for 4 cycles, then 4 more cycles every 3 months for maintenance	Peripheral neuropathy, reversible in 61% of patients
DRC^21^	Dexamethasone 20 mg IV on day 1 Rituximab 375 mg/m^2^ IV on day 1 Cyclophosphamide 100 mg/m^2^ oral twice daily on days 1–5 Repeat for 6 cycles	9% grade 3–4 neutropenia, 20% rituximab-associated toxicity
FCR^22^	Fludarabine 25 mg/m^2^ IV days 1–3 Cyclophosphamide 250 mg/m^2^ IV days 1–3 Rituximab 375 mg/m^2^ IV day 1 Repeat 28-day cycle × 4–6 cycles	45% grade 3–4 neutropenia
Fludarabine/rituximab^23^	Fludarabine 25 mg/m^2^ IV days 1–5 × 6 cycles Rituximab 375 mg/m^2^ IV on day 1 × 8 infusions	63% grade 3 or higher neutropenia 16% grade 3 or higher thrombocytopenia 14% pneumonia
Rituximab/cladribine^24^	Rituximab 375 mg/m^2^ IV on day 1 Cladribine 0.1 mg/kg subcutaneously days 1–5 Repeat monthly × 4 cycles	55% anemia 21% neurologic symptoms 14% symptomatic cryoglobulinemia 10% thrombocytopenia
CaRD^25^	Carfilzomib 20 mg/m^2^ IV in cycle 1, then 36 mg/m^2^ for cycles 2–6 Dexamethasone 20 mg IV on days 1, 2, 8, 9 Rituximab 375 mg/m^2^ IV on days 2 and 9 Maintenance: Carfilzomib 36 mg/m^2^ IV on days 1 and 2 Dexamethasone 20 mg IV on days 1 and 2 Rituximab 375 mg/m^2^ IV on day 2 Every 8 weeks for 8 cycles	77.4% hyperglycemia 41.9% hyperlipasemia
		
Ibrutinib^26^	420 mg oral once daily	Cytopenias, gastrointestinal symptoms, fatigue
Thalidomide/rituximab^27^	Thalidomide 50–200 mg/day oral daily days 1–28 Rituximab 375 mg/m^2^ IV weekly in weeks 2–5 and 13–16 28-day cycle × 12 cycles	44% grade 2 or higher peripheral neuropathy

Abbreviations: BoRd, bortezomib, rituximab, dexamethasone; BR, bendamustine, rituximab; CaRD, carfilzomib, cyclophosphamide, dexamethasone; DRC, dexamethasone, rituximab, cyclophosphamide; FCR, fludarabine, cyclophosphamide, rituximab; IV, intravenous; R-CHOP, Rituxan, cyclophosphamide, doxorubicin, vincristine, prednisone.

a Bortezomib and dexamethasone doses modified based on experience in myeloma to reduce neuropathy rates and other toxicities. After initial response, bortezomib and dexamethasone can be given weekly for 3 weeks and 1 week off.
